# The interaction of inflammasomes and gut microbiota: novel therapeutic insights

**DOI:** 10.1186/s12964-024-01504-1

**Published:** 2024-04-02

**Authors:** Shirin Manshouri, Farhad Seif, Monireh Kamali, Mohammad Ali Bahar, Arshideh Mashayekh, Rasol Molatefi

**Affiliations:** 1 Rajaei Cardiovascular Medical and Research Center, Iran University of Medical Sciences, Valiasr St, Niayesh Intersection, Tehran, 1995614331 Iran; 2 Department of Photodynamic Therapy, Medical Laser Research Center, Academic Center for Education, Culture, and Research (ACECR), Tehran, Iran; 3 Department of Immunology and Allergy, Academic Center for Education, Culture, and Research (ACECR), Tehran, Iran; 4 Department of Immunology, Medical School, Iran University of Medical Sciences, Tehran, Iran; 5 Cancer Immunology and Immunotherapy Research Center, Ardabil University of Medical Sciences, Ardabil, Iran; 6 Pediatric Department of Bou Ali Hospital, Ardabil University of Medical Sciences, Ardabil, 56189-85991 Iran

**Keywords:** Inflammasome, Gut microbiota, Dysregulation, Canonical, Non-canonical

## Abstract

Inflammasomes are complex platforms for the cleavage and release of inactivated IL-1β and IL-18 cytokines that trigger inflammatory responses against damage-associated molecular patterns (DAMPs) or pathogen-associated molecular patterns (PAMPs). Gut microbiota plays a pivotal role in maintaining gut homeostasis. Inflammasome activation needs to be tightly regulated to limit aberrant activation and bystander damage to the host cells. Several types of inflammasomes, including Node-like receptor protein family (e.g., NLRP1, NLRP3, NLRP6, NLRP12, NLRC4), PYHIN family, and pyrin inflammasomes, interact with gut microbiota to maintain gut homeostasis. This review discusses the current understanding of how inflammasomes and microbiota interact, and how this interaction impacts human health. Additionally, we introduce novel biologics and antagonists, such as inhibitors of IL-1β and inflammasomes, as therapeutic strategies for treating gastrointestinal disorders when inflammasomes are dysregulated or the composition of gut microbiota changes.

## Background

The mucosal lining of the gut tract develops physical barriers and immune defense systems to protect against microbial invasion. When there is an infection or cellular damage, the body responds through inflammation to maintain epithelial integrity [1]. It eradicates pathogens or repairs damaged cells or tissues. Innate immunity initiates recruitment of adaptive immune cells. The innate immune system contains a variety of germ-line-encoded pattern-recognition receptors (PRRs) that can detect microbial antigens, called pathogen-associated molecular patterns (PAMPs) or damage-associated molecular patterns (DAMPs), generated by cellular injury or tissue damage [2]. These PRRs include Toll-like receptors (TLRs) and C-type lectins (CLRs) located on the cell membrane as well as intracellular PRRs such as RIG-like receptors (RLRs) and DNA sensors such as Absent in melanoma 2 (AIM2). NOD-like receptors (NLRs) are other PRRs that can recognize molecular patterns derived from pathogens and damaged cells [3]. NLR family can be divided into three subfamilies: NODs, NLRPs, and IPAF. The NLR family in mammals contains three domains: a leucine-rich repeat (LRR) domain at the C-terminus, a nucleotide-binding NACHT domain in the central region, and a protein-protein interaction domain at the N-terminus composed of either a caspase activation and recruitment domain (CARD) or a Pyrin domain [4]. The NACHT acronym represents four proteins as the following: NAIP (neuronal apoptosis inhibitor protein), C2TA (MHC class 2 transcription activator), HET-E (incompatibility locus protein from Podospora anserina), and TP1 (telomerase-associated protein). The NACHT domain, which is a conserved protein domain across evolution, contains the NTPase domain and is present in both apoptotic proteins and those involved in MHC transcription [5].

**Fig. 1:**
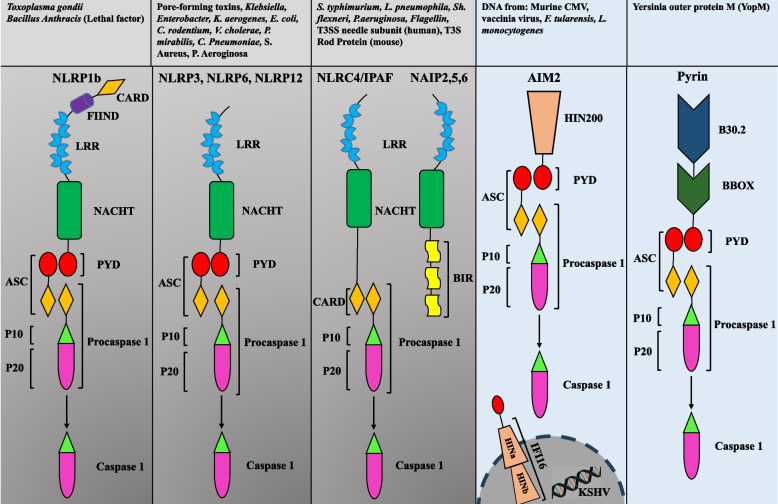
Structure of NLRP family, NLRC4/IPAF-NAIP family, PYHIN family, and pyrin inflammasomes. These cytosolic sensors contain various domains such as NACHT, LRR, PYD that interact with ASC to recruit and cleave pro-caspase-1 by CARD domain. Activated pro-caspase-1 cleaves inactivated pro-IL1β and pro-IL18 to release activated IL-1β and IL-18. NLRP1b can be activated by *T. gondii* and lethal factor of *Bacillus anthracis*. NLRP3 can be activated by pore-forming toxins, *Klebsiella, Enterobacter, K. aerogenes, E. coli, C. rodentium, V. cholerae, P. mirabilis, C. pneumoniae,* and *S. aureus*. NLRC4/IPAF-NAIP family inflammasome can be triggered by *S. typhimurium, L. pneumophila, Sh. flexneri, P. aeruginosa,* Flagellin, T3SS needle subunit (human), and T3S Rod Protein (mouse). AIM2 inflammasome is activated by Murine CMV, vaccinia virus, *F. tularensis,* and *L. monocytogenes.* IFI16 is specifically activated by Kaposi’s sarcoma-associated herpes virus (KSHV). Pyrin inflammasome is activated by Yersinia outer protein M (YopM). Abbreviations: Baculovirus inhibitor of apoptosis repeat domain (BIR); Caspase recruitment domain (CARD); function-to-find domain (FIIND); Leucine-rich repeat (LRR); Nucleotide-binding and oligomerization domain (NACHT); Pyrin domain (PYD)

Inflammasomes are intracellular multimeric protein complexes that are guardians of cellular integrity and control the integrity of various crucial cellular functions [6]. They form large multiprotein signaling platforms to cleave and activate caspase-1 (in the canonical pathway), which is a major inflammatory pathway. In the non-canonical pathway, cleavage of caspase-11 in mice (human orthologs include caspases 4 and 5) activates NLRP3 inflammasome which plays a pivotal role in the maintenance of intestinal immune homeostasis [7]. Active caspase-1 can cleave inactive forms of the pro-inflammatory cytokines of interleukin-1 beta (IL-1β) and IL-18 into their active forms [8]. Furthermore, inflammasome activation is linked to pyroptosis, a specific form of cell death mediated by gasdermin family proteins [9]. With the exception of AIM2, other inflammasomes such as NLRP1, NLRP3, and NLRC4 belong to NLRs Fig. 1 [10]. Various endogenous and exogenous stimuli have been shown to activate the inflammasome. Because numerous microbes colonize mucosal surfaces, maintaining homeostasis between the human body and microbiota necessitates a symbiotic interaction between them, which may cause different diseases if dysregulated. Inflammasome activation needs to be tightly regulated to limit aberrant activation and bystander damage to the host cells. Dysregulated inflammasome activity is associated with several inflammatory diseases, including autoimmunity, cancer, and gastrointestinal (GI) disorders [11]. Although inflammasomes primarily serve as intricate sensors, enabling the host to differentiate between beneficial and harmful bacteria, they also act as mediators in the communication between the host and its intestinal microbiota. Moreover, the environmental state of the intestinal lumen continuously influences the host response, leading to the generation of specific signals through the production of IL-1β or IL-18, which in turn modulates the intestinal microbiota. Subsequently, the modulated microbiota may enhance the host response through microbial by-products like short-chain fatty acids and bile acid derivatives. Therefore, inflammasomes are indispensable in orchestrating a precise reciprocal interaction within the body [12]. In this regard, investigation of the appropriate regulation of inflammasome activity and therapeutic interventions, by targeting structures related to inflammasome signaling, may be a promising area of research [13]. This review aims to discuss the interaction between inflammasomes and gut microbiota. In addition, we introduce novel biologics and antagonists as therapeutic strategies for GI disorders when inflammasomes are dysregulated or the composition of gut microbiota changes.

## Types of inflammasomes

### NLRP1 inflammasome

Although NLRP1 is the first reported molecule to form an inflammasome with minimal requirement of caspase-1, caspase-5, and ASC, its mechanism of activation remains poorly understood [14]. NLRP1 structurally differs from other NLRs in its additional C-terminal extension, consisting of a domain with an unknown function (function to find domain, FIIND) and a CARD domain. Human NLRP1 forms an ASC-dependent inflammasome, whereas mouse NLRP1 may activate caspase-1 in an ASC-independent manner [15]. NLRP1 inflammasome is different in mice and humans. Mouse NLRP1 inflammasome consists of three paralogs of Nlrp1a, b, and c, which contain an NR100 domain instead of the PYD seen in humans. Mouse NLRP1 can directly activate caspase-1 and ASC is not needed. In contrast, human NLRP1 inflammasome contains the N-terminal PYD. Its function is dependent upon ASC associated with the C-terminal CARD domain [16]. The best-characterized elicitor of NLRP1 activation is anthrax lethal toxin (LT), a major virulence factor of *Bacillus anthracis* [17]. Lethal factor (LF) is a zinc metalloprotease that uses a channel formed by the protective antigen to translocate into the cell cytosol, where it inactivates immune signaling by cleaving mitogen-activated protein kinase kinases. Interestingly, the protease activity of LF is also necessary to activate Nlrp1b, as inactive LF mutants do not trigger inflammasome assembly [18]. Its protective antigen (PA) subunit allows LF to enter the cytosol. LF activates caspase-1 and induces rapid cell death via NLRP1 in rats and NLRP1b in mice. Since inactive but structurally virtually identical mutants of LF fail to activate caspase-1, it is likely that LF does not directly bind to NLRP1 [17]. This hypothesis was confirmed by several studies that demonstrated that LF protease cleaves NLRP1 from the N-terminus in rat NLRP1 and mouse NLRP1b [19, 20]. LF can cleave cytosolic substrates, a process that additionally requires Ca^2+^ flux and probably proteasome activity [21]. Some evidence also suggests that NLRP1 undergoes autoproteolytic cleavage at a conserved motif within its FIIND [22] and that direct cleavage of a conserved motif in the FIIND by LF may present a necessary but not sufficient step for NLRP1 activation [23]. Activation of human and mouse NLRP1 also requires an autoprocessing event in a unique FIIND that lies between the C-terminal CARD and LRR motifs; however, this process is not carried out by LF [24]. Moreover, murine Nlrp1b is highly polymorphic, and different mouse strain variants of Nlrp1b confer susceptibility to LF-induced caspase-1 activation [25]. Nlrp1b activation by LT represents an essential host defense mechanism for the control of *B. anthracis* infection [26]. A hyperactive mutation in the murine Nlrp1a paralog triggers a systemic caspase-1–dependent inflammatory response in vivo [27]. IL-1β is critical for multiorgan neutrophilic disease, whereas ASC is indispensable for the inflammatory phenotype. The exact Nlrp1a ligand or activation signal needs to be elucidated, but studies in Nlrp1a deficient mice suggest a role for Nlrp1a in triggering pyroptosis in hematopoietic progenitor cells during periods of hematopoietic stress induced by chemotherapy or infection [27, 28]. Nevertheless, it is now accepted that rodent NLRP1 proteins recognize anthrax toxin by acting as decoy substrates for the LF protease. A similar decoy mechanism has also been reported for certain plant resistance gene products, for example, Arabidopsis PBL2, which is uridylated and thus activated to drive immunity by the *Xanthomonas campestris* effector AvrAC, which normally dampens plant immune resistance by uridylating and inactivating BIK1 kinase. Importantly, this mechanism limits the evasion of immune recognition because any evasion strategy would require changing the substrate specificity of the pathogen effector proteins, and consequently result in a loss of its primary target activity [29]. Tye et al. demonstrated that the NLRP1 inflammasome could control the gut microbiota of littermate control mice [30]. Nlrp1-deficient mice showed an increased number of bacteria that produce butyrate. They belong to the order Clostridiales and protect against DSS-induced colitis. Butyrate has been proven to have beneficial effects on Inflammatory bowel disease (IBD) by promoting the functions of the intestinal barrier, such as mucus production and tight junctions [12]. Thus, the NLRP1 inflammasome may negatively affect IBD by decreasing butyrate production by the gut microbiota. IBD is an autoimmune disorder with two major clinical forms, including Crohn’s disease and ulcerative colitis. Short-chain fatty acids (SCFAs) are generated by beneficial gut bacteria through the fermentation of a diet high in fiber, which the body cannot directly digest. These SCFAs play a crucial role in reducing inflammation, regulating immune function, and preventing an overactive immune response, thereby slowing down the clinical progression of IBD [31].

#### Direct and indirect activators

Direct activators of the NLRP1 inflammasome include LT, which can activate macrophages with certain mouse *Nlrp1b* or rat *Nlrp1* alleles, and IpaH7.8 E3 ubiquitin ligase secreted by *S. flexneri*, which can ubiquitinate and activate mouse *Nlrp1b* allele 1 [32]. The FIIND domain of NLRP1 undergoes posttranslational autoproteolysis, producing C-terminal and N-terminal fragments that associate via noncovalent bonding. Proteolytic cleavage of NLRP1 by LT or enteroviral 3C protease results in liberation of the C-terminal fragment and subsequent assembly and activation of pro-caspase-1, leading to pyroptosis. The N-end rule E3 ligase UBR2 plays a role in recognizing and ubiquitinating the neo-N-terminus of NLRP1, while cleavage of the FIIND domain blocks degradation of the C-terminal fragment. This model suggests that pathogens such *S. flexneri* destroy host NLR proteins and the N-terminus of NLRP1 to induce an immune response, and provides an explanation for the high polymorphism of NLRP1 [33, 34]. However, more research is needed to validate the decoy model and the hypothesis that pathogen effectors also destroy the intended targets, such as other NLR proteins.

Indirect activators of the NLRP1 inflammasome include *Toxoplasma gondii*, VbP, and metabolic inhibitors, such as 2-deoxyglucose and sodium azide, an oxidative phosphorylation inhibitor, particularly in HT1080 cells [35]. NLRP1b can be activated in RAW 264.7 cells upon encounter with *Listeria monocytogenes* or *S. flexneri* [36]. Knockdown of NLRP1 by short interfering RNA (siRNA) in Lewis rat bone marrow-derived macrophages decreased cell death caused by *T. gondii*, whereas overexpression of Nlrp1 allele 5 in CDF macrophages had the opposite effect, indicating a close relationship between *T. gondii*-induced pyroptosis and NLRP1 [37]. However, shRNA knockdown of NLRP1 in human MonoMac6 cells increased cell death caused by T. gondii, contradicting the role of NLRP1 in rats. VbP stimulates both mouse and rat NLRP1 inflammasomes and can modify hNLRP1 by degrading the N-termini of sensitive PRRs. This results in the liberation of C-terminal fragments and establishment of inflammasomes, but does not directly cleave the N-terminal fragments, suggesting that pyroptosis caused by NLRP1 is independent of the N-end rule pathway [38]. Human DPP9 inhibits NLRP1 activation by associating with FIIND. Indirect activators sense disturbances in cellular homeostasis and metabolism, leading to E3 ligase-mediated degradation of the N-terminus of NLRP1 and subsequent activation. However, the detailed mechanisms of the cell perturbations induced by these indirect activators remain to be identified, and the specific relationships between T. gondii, VbP, and metabolic inhibitors have not been reported. In contrast to direct stimuli, pyroptosis induced by indirect stimuli is independent of the direct cleavage of the N-terminal fragment [39].

### NLRP3 inflammasome

The NOD-like receptor protein-3 (NLRP3) inflammasome includes a pyrin domain (PYD) at the N-terminus, a central NACHT domain (including seven motifs with a nucleotide adenosine triphosphate/guanosine triphosphate (ATP/GTPase) P-loop and Walker A and B binding sites), and nine leucine-rich repeats (LRR) at the C-terminal Fig. 1 [40]. Although NLRP3 is inactive in its monomeric form, its assembly results in NLRP3 inflammasome formation through interactions with apoptosis-associated speck-like protein containing a caspase recruitment domain (CARD) (ASC) [41]. The NACHT domain is pivotal for NLRP3 activation and self-assembly. ASC initiates NLRP3 oligomerization, which recruits pro-caspase-1 (inactive form). The cleavage of pro-caspase-1 leads to the release of active caspase-1. It binds to the ASC CARD domain [42]. NLRP3-ASC-pro-caspase-1 complexes form an inflammasome with a ring-like structure. Similar to the NLRC4 disk structure, the NLRP3 inflammasome may be composed of 11 subunits. Subsequently, caspase-1 cleaves two inactive pro-inflammatory cytokines, pro-interleukin 1-beta (pro-IL-1β) and pro-interleukin 18 (pro-IL-18), into active forms of IL-1β and IL-18 [43].

In contrast to other inflammasomes, NLRP3 inflammasome requires two signals. Signal 1 (priming) is supplied by microbial molecules or the activation of endogenous cytokines or PRRs, such as TLRs, leading to the transcriptional upregulation of canonical and non-canonical NLRP3 inflammasome components, as described below [44]. It consists of transcriptional upregulation of NLRP3 and pro-IL-1β, and non-transcriptional mechanisms such as dephosphorylation of residues within the N-terminal PYD [45], phosphorylation of a critical serine residue between the PYD and NACHT domains [46], and NLRP3 deubiquitination [47]. Caspase-8 and FAS-associated death domain protein (FADD) mediate this step by regulating the NF-kB pathway. Lys-63-specific deubiquitinase BRCC36 (BRCC3) and IL-1 receptor-associated kinase 1 (IRAK1) regulate the activation of NLRP3 [48]. Signal 2 (activation) is supplied by either PAMPs or DAMPs, pore-forming toxins, K^+^ efflux, lysosomal disruption, mitochondrial reactive oxygen species production, relocalization of cardiolipin to the outer mitochondrial membrane, and the release of oxidized mitochondrial DNA, followed by Cl^−^ efflux [49]. *Chlamydia pneumoniae*, a common respiratory pathogen involved in atypical pneumonia, activates NLRP3 and induces IL-1β secretion by macrophages [50]. In addition, several pore-forming, bacterial toxins, such as valinomycin (some Streptomyces strains), nigericin (*Streptomyces hygroscopicus*), aerolysin (*Aeromonas hydrophila*), maitotoxin (*Gambierdiscus toxicus*), and listeriolysin O (*L. monocytogenes*) induce NLRP3 inflammasome assembly [51]. Nigericin is a potassium ionophore. It activates NLRP3 by K^+^ efflux because it facilitates H^+^/K^+^ anti-port across cell membranes. Armstrong et al. conducted an in vitro study. They examined the effects nigericin on Raw 264.7 macrophages, which lack PYCARD/ASC and were unable to activate NLRP3 inflammasome. They indicated that nigericin, as NLRP3 agonist, could enhance killing of *C. rodentium*, and initiate inflammation through pathways unassociated with the NLRP3 inflammasome, independent of ASC. This point suggests new methods to change the immune response conditions in IBD [52].

Moreover, Ca^2+^ flux is suggested to cause mitochondrial dysfunction and is involved in NLRP3 inflammasome activation. Viral RNAs activate NLRP3 via mitochondrial antiviral signaling proteins (MAVs) located on the outer membrane of mitochondria [53]. Mitochondrial dysfunction plays an important role in inflammasome activation. Upon receiving certain signals, mitochondrial DNA is released and binds to the NLRP3 inflammasome, leading to its activation. However, the exact interactions and functions of other proteins involved in this process, such as MAVS and Mitofusion 2 (Mfn2), are not fully understood [54, 55]. Particulate matter activates NLRP3 through lysosomal rupture-induced K^+^ efflux and perhaps the release of cathepsins [56]. Some commensal gut microbes may activate NLRP3 inflammasome in macrophages of the intestinal mucosa. Seo et al. reported that *Proteus mirabilis* might be an NLRP3 activator via hemolysin production [57]. Kitamoto et al. indicated that Enterobacter and Klebsiella spp. Colonization of the oral cavity may trigger the NLRP3 inflammasome. In this regard, *K. aerogenes* residing in the oral cavity of mice results in periodontitis by secretion of IL-1β from macrophages [58]. In contrast, several studies have reported that Nlrp3-deficient mice are susceptible to DSS-induced colitis owing to IL-18 overproduction [59]. However, Bauer et al. indicated that macrophages incubated with dextran sulphate sodium (DSS) in vitro produce high concentrations of IL-1β via a caspase 1-dependent pathway. They also showed that DSS did not activate caspase 1 in macrophages lacking NLRP3 [60]. Zhang et al. reported a causal effect of aberrant accumulation of gut microbiota on age-related atrial fibrillation, indicating that the microbiota-intestinal barrier-atrial NLRP3 inflammasome interaction may serve as a potential target for the treatment of age-related arrhythmia [61]. New evidence has revealed how *Staphylococcus aureus* evades macrophage killing, suggesting a new target for the development of antibiotics. In preclinical studies, researchers found that *S. aureus* sequesters mitochondria away from phagosomes to evade bactericidal reactive oxygen species (ROS). This evasion of macrophage death is dependent on the NLRP3 inflammasome [62]. Researchers found that targeting NLRP3 using a small interfering RNA (siRNA) improved bacterial clearance in mice infected with *S. aureus*. Researchers also discovered that a combination of NLRP3 inhibition and electron transport chain complex II inhibition improved the killing of *S. aureus* in human monocytes [63]. Recently, White et al. demonstrated the activation of the NLRP3 inflammasome following cigarette smoke exposure, which primes the lungs for acute injury caused by *Pseudomonas aeruginosa*. This study suggested that targeting the NLRP3 inflammasome may be a potential therapeutic approach for treating cigarette smoke-induced lung injury [64].

#### Mechanisms of canonical, non-canonical and alternative activation of NLRP3

**Fig. 2:**
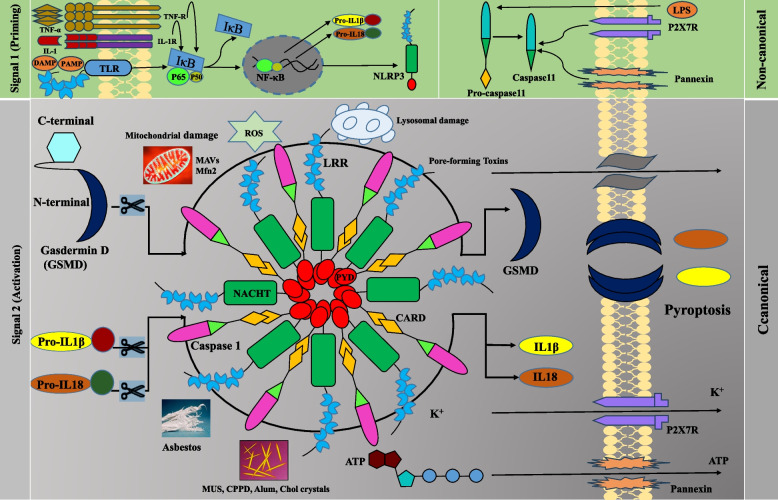
Mechanisms of canonical and non-canonical activation of NLRP3. The canonical pathway involves TLR signaling, which induces the transcription of IL-1β, IL-18, and NLRP3 through NF-κB pathway. The non-canonical pathway involves stimuli such as LPS, which require caspase-11 for caspase-1 activation. Upon sensing danger signals, NLRP3 assembles into a multiprotein complex with the adaptor protein ASC and recruits caspase-1. Activated caspase-1 cleaves pro-IL-1β and pro-IL-18 into their mature forms, which are then secreted to initiate an immune response. Non-canonical pathway of TLR signaling for NLRP3 involves the activation of caspase-11 by cytosolic LPS from Gram-negative bacteria. Caspase-11 activation leads to the cleavage of gasdermin D, which forms pores in the cell membrane and triggers pyroptosis. Caspase-11 activation also induces the opening of the pannexin-1 channel, which leads to K+ efflux and NLRP3 inflammasome activation. The activation of P2X7 by ATP released from the pannexin-1 channel also contributes to pyroptosis. Signal 1 (priming) involves the transcriptional upregulation of NLRP3, pro-IL-1β, and pro-IL-18 by TLR, TNFR, and IL-1R signaling pathways. Signal 2 (activation) results in the cleavage and release of mature IL-1β and IL-18. It can be triggered by various stimuli, including extracellular ATP, pore-forming toxins, K+ efflux, lysosomal and mitochondrial damage, crystal deposition such as monosodium urate (MSU), Calcium pyrophosphate deposition (CPPD), alum, and cholesterol crystals, and reactive oxygen species (ROS)

TLR signaling induces IL-1β and NLRP3 transcription. In contrast, NLRP3 can be primed by a TLR-dependent pathway but in a transcription-independent signaling manner. Activated NLRP3 forms a multiprotein structure with adaptor protein ASC and recruits caspase-1. Subsequently, activated caspase-1 cleaves pro-IL-1β and pro-IL-18, which are secreted [65]. A common mechanism or signaling mediator for canonical NLRP3 activation has not been identified; however, many models, such as ROS production or Ca^2+^ release from the endoplasmic reticulum, have been suggested. In contrast, non-canonical stimuli such as lipopolysaccharide (LPS) of gram-negative bacteria such as *Escherichia coli, Citrobacter rodentium,* and *Vibrio cholerae* require caspase-11 for the complete activation of caspase-1 [66]. The transcription of caspase-11 is regulated by the type I IFN signaling pathway, which is concomitantly induced by TLR signaling. Although the molecules linking caspase-11 to caspase-1 have not been fully detected [67]. It has been proposed that active caspase-11 induces the opening of the pannexin- 1 channel through cleavage, which initiates ATP release for NLRP3 inflammasome activation. Priming the caspase-11 pathway in vivo with LPS or a Toll-like receptor-3 (TLR3) agonist led to high mortality in wild-type (WT) mice after secondary LPS exposure, but not in Casp11(−/−), Panx1(−/−), or P2 × 7 (−/−) mice. Caspase-11 activation initiates pyroptosis via the cleavage of gasdermin D (GSDMD) [68]. Active caspase-1 and caspase-11 cleave GSDMD into two fragments, including the N and C domains, after an aspartic acid residue at position 276 (human) or 275 (mouse). The GSDMD-N domain can form pores in the membrane of the target cell [69]. P2X7 activation by ATP released from the pannexin-1 channel facilitates extracellular release of ATP, which may lead to pyroptosis [70]. Alternative activation of the NLRP3 inflammasome by LPS occurs in human monocytes. Here, K^+^ efflux is dispensable, RIPK1, FADD, and CASP8 are involved, and no pyroptosis is induced [71] (Fig. 2).

### NLRP6 inflammasome

NLRP6, also known as PYPAF5, was first described as a modulator of NF-κB and caspase-1 expression in most immune cells. This protein is found in murine intestinal epithelial cells such as enterocytes. Studies propose that NLRP6 is essential for regulating the composition and function of the intestinal microbiome [72]. It also orchestrates interactions of the host with enteric viral and bacterial infections through inflammasome-dependent and inflammasome-independent pathways, colitis-associated tumorigenesis, and mucus secretion in goblet cells [73]. NLRP6 deficiency in intestinal epithelial cells is linked to disrupted IL-18 production and caspase-1 activation. NLRP6-deficient mice show an outgrowth of Prevotellaceae and TM7, and reductions in Lactobacilli and Firmicutes [74]. These imbalances trigger predisposition to colitis and spontaneous inflammation in the intestine. Microbiota-associated metabolites, such as taurine and commensal bacteria in the intestine, can activate NLRP6 inflammasome to produce antimicrobial peptides [75]. Goblet cells can activate the NLRP6 inflammasome through TLR-Myd88 signaling, resulting in muc2 production. Gram-positive pathogens produce lipoteichoic acid, which activates the NLRP6 inflammasome through ASC recruitment, leading to systemic infection. Stress-induced corticotropin-releasing hormone (CRH) inhibits activation of the NLRP6 inflammasome, which causes intestinal inflammation and alterations in the gut microbiome [76]. NLRP6-deficient mice are susceptible to inflammation. However, further investigations are required to identify additional activators and negative regulators of the NLRP6 inflammasome and mechanisms of modulation in GI inflammasome-associated diseases [13]. The NLRP6 inflammasome was autoinhibited under normal conditions. Lipoteichoic acid and double-stranded RNA (dsRNA) can bind directly to NLRP6, thereby making a possible conformational change to help liquid-liquid phase separation (LLPS) an early step essential for inflammasome assembly [77]. Moreover, LPS can bind directly to NLRP6, which may lead to the formation of LLPS. Then, its interaction with ASC activates caspase-1 and/or caspase-11, which activates GSDMD and leads to pore formation in the plasma membrane and the release of proinflammatory cytokines and intracellular contents. If the interaction with ASC does not form the NLRP6 inflammasome, NLRP6 in LLPS induces an alternative inflammasome-independent pathway by inducing interferon (IFN)- and IFN-stimulated genes [78]. Altogether, NLRP6 may play a protective role via the TLR axis under conditions where strong inflammatory responses are destructive, while its action is necessary for the maintenance of homeostasis in the intestine [79]. The NLRP6 inflammasome is linked to homeostasis of the GI tract. Previous studies have demonstrated that NLRP6 dysregulation may lead to GI dysbiosis [80]. Moreover, NLRP6 inflammasome stimulates the expression of antimicrobial peptides (AMPs), including angiogenin-4 (Ang4). Some microbial metabolites, including taurine, spermine, and histamine, appear to induce NLRP6-dependent production of IL-18 and AMPs [75]. Finally, defects in NLRP6 activation lead to autophagy dysfunction [81]. Seregin et al. reported that IL-18 production was highly dependent on NLRP6 activation. Both Il18 −/− and Il18r1 −/− mice showed an accumulation of *A. muciniphila*; thus, the use of recombinant IL-18 decreased the amount of *A. muciniphila* in Nlrp6 −/− mice [82].

### NLRP12 inflammasome

NLRP12, also known as Nalp12 and Pypaf-7, forms an inflammasome with ASC and caspase-1 to mature IL-1β. It was one of the first NLRs to colocalize and interact with the adaptor protein ASC to form an inflammasome. Mutations in NLRP12 coding sequence in the human genome are associated with IL-1-mediated inflammatory diseases [83]. Although our understanding of the role of NLRP12 in health and disease is limited, recent data suggests that NLRP12 is crucial for the recognition of *Yersinia pestis*, the causative agent of plague. NLRP12 controls caspase-1 cleavage and IL-1β and IL-18 secretion upon *Y. pestis* infection by macrophages [84]. However, NLRP12 can inhibit the production of IL-12 by bone marrow-derived macrophages and negatively regulate host defense against *Brucella abortus* [85]. The exact ligand of NLRP12 is currently unknown; however, its activation requires a functional T3S system. This suggests that a bacterial virulence factor gaining access to the host cytosol may be necessary to directly activate NLRP12 or alter host signaling pathways. Regardless of the activation mechanism, NLRP12-driven IL-18 secretion and associated IFN-γ production play a critical role in resistance against *Y. pestis* infection in mice; NLRP12-deficient mice showed higher mortality and bacterial loads after infection [86]. In addition to forming an inflammasome, NLRP12 plays a role in suppressing intestinal inflammation and tumorigenesis by negatively regulating NF-kB signaling [87]. Several independent studies have shown that NLRP12 negatively regulates both canonical and non-canonical NF-κB signaling in biochemical assays, colon cancer, and colitis models [88]. NLRP12 was suggested to inhibit host defense independent of the inflammasome during *Salmonella enterica serovar Typhimurium* infection, as Nlrp12-deficient mice were more resistant to *S. typhimurium* infection than WT controls and presented lower inflammatory cytokine levels [89]. NLRP12 functions in hematopoietic cells to suppress tumorigenesis, but it is not hematopoietic but the non-hematopoietic compartment, which is central to limiting tumor numbers. Nonetheless, both studies suggest an important role of NLRP12 in controlling inflammatory responses in the colon [90].

### NLRC4 inflammasome

NLRC4 (formerly known as IPAF, Card12) can form an inflammasome following infection with various gram-negative bacteria, such as *S. typhimurium*, *Legionella pneumophila*, *Shigella flexneri*, and *Pseudomonas aeruginosa*. NLRC4 is expressed in the myeloid lineage, and its fundamental role is to protect against bacterial invasion. Unlike other inflammasomes, NLRC4 is activated in association with another NLR protein, NAIP, which serves as a receptor for NLRC4 activators [91]. The NAIPs and NLRC4 share two common domains. The NACHT domain consists of a nucleotide-binding domain (NBD), helical domain 1 (HD1), winged-helix domain (WHD), helical domain 2 (HD2), and a C-terminal LRR [92]. Furthermore, NAIP has three Baculovirus Inhibitor-of-Apoptosis repeat (BIR) domains, and NLRC4 contains an N-terminal CARD. The NBD and WHD interactions are crucial for maintaining NLRC4 in an auto-inhibited state [92]. Although only one NAIP is expressed in humans, which can bind to *Chromobacterium violaceum* needle protein, Cprl-, there are seven NAIP genes in C57BL/6 mice, among which only four NAIPs are expressed, including NAIP1, which can bind to needle proteins of the type III secretion system, NAIP2 to the Salmonella SPI-1 basal rod component PrgJ, and NAIP5 and NAIP6 to flagellin [93]. The only known human NAIP is activated by flagellin or Cprl, but not by the T3SS rod protein (binds to mouse NAIP2) or PrgJ. It then binds to the T3SS needle subunit [94]. It appears that bacterial type III or type IV secretion systems are required for caspase-1 cleavage in macrophages. However, some strains deficient in flagellin, such as *S. typhimurium* and *L. pneumophila,* are defective in inducing NLRC4. It has also been reported that NK cells secrete IFN-γ against *S. typhimurium* by flagellin-sensing NLRC4 inflammasomes [95]. Several studies have shown that NLRC4 is effective in host defense against flagellated *Pseudomonas aeruginosa*. In the absence of NLRC4 or caspase-1 activation, Legionella-containing phagosomes cannot fuse with lysosomes. In contrast, flagellin-mutated Legionella cannot activate caspase-1 in macrophages [96]. Man et al. revealed that *S. typhimurium* can activate NLRC4 and NLRP3, which leads to ASC formation and recruitment of caspases to the inflammasome. The NLRC4 inflammasome senses PrgJ, a component of the type III secretion system (T3SS), and initiates inflammasome assembly through CARD-CARD interactions with caspase-1 [97]. Researchers have demonstrated that gain-of-function mutations in *nlrc4* are associated with an extremely rare disease called autoinflammation with infantile enterocolitis (AIFEC). This disease is characterized by activation of macrophages and severe inflammation of the GI tract [98]. Interestingly, resident intestinal mononuclear phagocytes (iMPs), such as dendritic cells and macrophages, combat the gut pathogenic microbiota while maintaining tolerance to commensal microbes. Because immune cells of the GI tract largely react with many commensal microbes, they apply several mechanisms to limit uncontrolled immune responses against GI commensals. Upon NLRC-4 activation in iMPs, IL-1β is secreted to induce the expression of adhesion molecules in the endothelium. These adhesion molecules facilitate the recruitment of neutrophils to the intestinal mucosa and ingestion of foreign microbes [99].

### PYHIN inflammasomes

Another class of inflammasomes, distinct from NLRs, has been identified that contains members of the PYHIN family. PYHIN proteins are encoded by a family of four human genes (AIM2, IFI16, MNDA, and IFIX) and 13 mouse genes, and contain a PYD and one or two HIN-200 DNA-binding domains. AIM2 and IFI16 have been shown to form caspase-1-activating inflammasomes. Unlike NLRs, AIM2 and IFI16 bind directly to their ligand, dsDNA, in both cases. ASC is required for the recruitment of pro-caspase-1 because AIM2 and IFI16 lack CARDs [100]. AIM2 is located in the cytosol and senses dsDNA of a viral or bacterial origin. The recognition of dsDNA by AIM2 depends on the length of the DNA rather than its sequence. However, dsDNA less than 80 bp in length is a poor activator of the AIM2 inflammasome. AIM2 can recognize self-DNA and activate inflammasome complexes, but its cytosolic location limits the recognition of self-DNA under steady-state conditions [101]. Nonetheless, in situations where self-DNA is inefficiently cleared from the extracellular milieu or improperly degraded in the phagolysosomal compartment, it can access cytosolic compartments, overexpress genes encoding type I IFNs and associated genes, and activate AIM2 to drive inflammation [102]. During infection, AIM2 senses DNA from murine cytomegalovirus, vaccinia virus, *Francisella tularensis*, and *L. monocytogenes*. The function of AIM2 inflammasome is to modulate gut microbiota. Hu et al. showed that the activation of the AIM2 inflammasome results in the production of IL-18 and AMPs in the gut [103]. Aim2-deficient mice show a reduction in IL-18 and AMPs, such as REG3c and REG3b. The paucity of AIM2 leads to gut dysbiosis, thus increasing the susceptibility to DSS-induced colitis. They revealed that the number of Enterobacteriaceae family members, such as *E. coli*, in the feces of Aim2-deficient mice was 1000-fold higher than that in WT mice. When *F. novicida*, a cytosolic pathogen, escapes the vacuole into the cytoplasm, the AIM2 inflammasome is stimulated. *F. novicida* mutants lacking critical genes for escaping the vacuole cannot trigger the AIM2 inflammasome [104]. In the GI tract, untreated Aim2 deficient mice indicated higher numbers of *Akkermansia muciniphila* and Anaeroplasma, and lower numbers of Bifidobacterium, Prevotella, Anaerostipes, Flexispira, and Paraprevotella species than in WT mice [105]. Similar to NLRP6, AIM2 inflammasome is essential for maintaining microbial homeostasis in the intestine [106]. IFI16 is mainly located in the nucleus of cells and detects viral dsDNA to drive the production of type I IFNs. However, IFI16 also recognizes the genome of *Kaposi’s sarcoma-associated herpes virus (KSHV)* in the nuclei of infected cells. In response to KSHV, IFI16 and ASC translocate from the nucleus to the cytosolic perinuclear region, where they form an inflammasome complex with caspase 1 [107]. It is not clear how IFI16 discriminates between self-and viral DNA in the nucleus, but one possibility is that self-DNA is inaccessible to IFI16 in the context of chromatin. Nonetheless, how this inaccessibility is maintained during cell division remains to be addressed [108].

### Pyrin inflammasome

The pyrin is a high-molecular-weight (86 kDa) protein primarily found in immune cells, including neutrophils, monocytes, and DCs. It comprises four functional domains: PYD, zinc finger domain (bBox), coiled-coil (CC) domain, and B30.2/SPRY domain. Unlike other immune sensors, pyrin detects bacterial virulence via cytoskeletal remodeling rather than microbial compounds [11]. Pyrin mediates caspase-1 inflammasome assembly in an ASC-dependent manner upon recognition of an inactivation modification of RhoA GTPase by pathogens. Mouse pyrin has two functional phosphorylation sites, Ser-205 and Ser-241, that render pyrin inactive by binding to 14-3-3 proteins. Upon toxin stimulation or bacterial infection, resulting in Rho modification, Ser-205 and Ser-241 are dephosphorylated, leading to 14-3-3 dissociation. This cascade results in activation of pyrin and formation of an oligomeric pyrin-ASC inflammasome complex [11]. Pathogenic Yersinia spp. secrete a virulence factor, Yersinia outer protein M (YopM), which suppresses pyrin inflammasome activation by employing the host kinases PRK1 and PRK2 to maintain pyrin in an inactive phosphorylated state [109]. Pyrin associates with cytoskeletal microtubules and actin filaments, and drugs interfering with microtubule dynamics inhibit pyrin inflammasome formation without affecting the pyrin phosphorylation state. This suggests that microtubule elements control the activation of the pyrin inflammasome, perhaps by impacting the conformational change of dephosphorylated pyrin and the associated ASC recruitment [110]. Although knowledge regarding the specific pyrin inflammasome activators produced by the residing intestinal microbiota is scarce, recent studies have shed light on their role in maintaining intestinal homeostasis. In a study employing DSS-induced colitis in mice, pyrin inflammasome signaling prevented dysbiosis, promoted intestinal barrier integrity, and ameliorated colonic inflammation and tumorigenesis. In a recent study using whole-genome pooled CRISPR screen technology, two bile acid analogs (BAA485 and BAA473) were identified as specific ligands that induce pyrin inflammasome signaling in myeloid and IEC lines [11]. As the enteric bacterial metagenome is a rich source of bile acid metabolism, similar microbiome-derived pyrin inflammasome activating ligands may contribute to the regulation of intestinal homeostasis; however, the existence of such pyrin-specific ligands has not yet been verified in situ. Overall, the pyrin inflammasome provides a new paradigm for innate immune components that engage with the cytoskeleton, offering new mechanisms for structural modulation of cellular immunity [111].

## Future clinical perspectives

Heat shock protein 90 (HSP90) and ubiquitin ligase-associated protein SGT are critical for NLRP3 activation. The interaction of these molecules with NLRP3 has been suggested to maintain NLRP3 in an inactive state. Once the signals are triggered, HSP90 and SGT1 dissociate from NLRP3, leading to inflammasome assembly. Downregulation of SGT1 by siRNA or chemical inhibition of HSP90 can prominently reduce inflammasome function [112]. Inhibition of cathepsin B following lysosomal damage using the pharmacological inhibitor CA074-Me significantly decreased IL-1β release and caspase-1 activation in murine macrophages [113]. MCC950/CRID3 is a selective NLRP3 inhibitor that can mitigate canonical and non-canonical activation of the NLRP3 inflammasome. A previous study has demonstrated the efficacy of MCC950 in the treatment of murine ulcerative colitis. This agent suppresses IL-1β and IL-18 at both translational and transcriptional levels. This finding broadens new horizons to a promising approach for the treatment of inflammatory bowel diseases such as colitis other than ulcerative colitis [114]. Jiao et al. reported that MCC950 reduced inflammation in a mouse model of spinal cord injury, leading to improvements in neurological outcomes in vitro and in vivo [115]. A recent study showed intracerebral hemorrhage can lead to gut microbiota dysbiosis in mice, and selective NLRP3 inflammasome inhibition by MCC950 could alleviate gut microbiota dysbiosis and neurobehavioral deficits [116]. Moreover, studies have revealed that MCC950 inhibits the secretion of IL-1β by suppressing the NLRP3 and AIM2 inflammasomes, but not the NLRC4 inflammasome. MCC950 may target glutathione S-transferase omega 1 (GSTO1) protein. This protein may also interact with the ASC domain [117]. Previous reports have indicated that the selective P2X7 antagonist A804598 caused a prominent increase in the contractions of bowel inflammation in a rat colitis model [118]. However, a recent study demonstrated that mice deficient in the P2X7 receptor exhibit defective innate immunity, resulting in weaker and incomplete inflammatory responses to T. gondii infection than their WT counterparts, resulting in ileal tissue disruption and parasite dissemination. In P2X7 deficiency, indigenous gut microbiota may create an environment that increases susceptibility to T. gondii-induced ileitis. In other words, P2X7 protects mice against T. gondii infection [119]. Therefore, further investigations are warranted to elucidate the final consequences of P2X7 purinergic antagonists. In addition, other antagonists such as Ac-YVAD-CHO, a polypeptide with a sequence homologous to special sequences of caspase substrates, which selectively inhibits caspase-1 activation, and a VX-765 inhibitor, known as belnacasan, which reduces the activity of caspase-1, should be taken into consideration as well; however, their clinical efficacy remains unclear [120].

The use of Anakinra, a recombinant form of interleukin-1 receptor antagonist (IL-1Ra), may be promising for treating GI problems. Liso et al. investigated the efficacy of Anakinra in treating patients with ulcerative colitis (UC) who did not respond to anti-tumor necrosis factor (TNF) therapy. Winnie-TNF-KO mice were used as a model for primary anti-TNF non-responders. They found that patients with UC who did not respond to anti-TNF therapy had high levels of IL-1β. Administering anakinra to mice effectively reduced inflammation in the colon and decreased the number of IFN-γ-expressing CD8^+^ T cells. This study suggests that targeting IL-1β may be a more effective therapeutic option for primary non-responders to anti-TNF therapy in UC patients [121]. Few infectious complications have been reported after administration of other IL-1 inhibitor biologics, such as rilonacept (a dimeric fusion protein consisting of the ligand-binding domains of the human IL-1R extracellular domains linked to the Fc portion of human IgG1), canakinumab (a fully humanized monoclonal antibody targeting IL-1β but not IL-1α), and gevokizumab (a monoclonal anti–IL-1β antibody that negatively affects IL-1β signaling through an allosteric mechanism). It reduces the binding affinity of IL-1β for IL-1R type I, but not for IL-1 decoy receptor type II (IL-1 receptor antagonist); however, further studies are needed to elucidate the interaction between the gut microbiota and inflammasomes [122].

## Conclusion

The interaction between inflammasomes and the gut microbiota plays a crucial role in maintaining gut homeostasis and regulating immune responses. Dysregulation of this interaction can lead to the development of various GI disorders. Therefore, it must be tightly regulated to limit aberrant activation and bystander damage to the host. Appropriate regulation of inflammasome activity and therapeutic interventions targeting structures related to inflammasome signaling may be promising areas of research. Inhibitors of the inflammasome and its downstream mediators such as IL-1β and IL-18 may be optimal targets for the treatment of various inflammatory diseases. Further research is needed to understand the precise mechanisms involved in inflammasome regulation and to identify novel compounds for pharmacological activation or inhibition of inflammasomes. We can develop new strategies to prevent and treat a wide range of human diseases associated with dysregulated inflammasome activity caused by the gut microbiota. However, inhibition of the inflammasome may have no effect or even a negative impact in individuals lacking increased expression of inflammasome-related pathways. Given the inflammasome’s ability to prevent the proliferation of harmful bacteria, inhibiting the inflammasome may worsen diseaseas. Therefore, identifying patients who may benefit from inflammasome-targeted therapies is crucial in a clinical setting.

## Data Availability

Please get in touch with the corresponding author for data requests.
